# Work-Related Dreams: An Online Survey

**DOI:** 10.3390/clockssleep2030021

**Published:** 2020-07-17

**Authors:** Michael Schredl, Lilian Marie Anderson, Lea Katharina Kahlert, Celine Sophie Kumpf

**Affiliations:** Central Institute of Mental Health, Medical Faculty Mannheim, Heidelberg University, Gesundheit, Postfach 12 21 20, 68072 Mannheim, Germany; landerso@mail.uni-mannheim.de (L.M.A.); lkahlert@mail.uni-mannheim.de (L.K.K.); cekumpf@mail.uni-mannheim.de (C.S.K.)

**Keywords:** work-related dreams, dream emotions, continuity hypothesis, gender differences

## Abstract

Professional work is an integral part of modern life. According to the continuity hypothesis of dreaming, which states that dreams reflect waking life, work-related dreams should be quite common. As most dream content analytic studies are carried out in student samples, the topic of work in dreams is understudied. A few small studies indicate that the stress levels associated with the job are especially reflected in work-related dreams. Here, a total of 1695 people (960 women, 735 men) completed an online survey that included questions about the estimated percentage of work-related dreams, the overall emotional tone of work-related dreams, and waking-life experiences related to their current job situation (working or not working). The findings indicate that every fifth dream is related to current or previous work. Individuals who are working dreamed more often about work, with jobs that are experienced as being more stressful being more likely to affect dream content. The emotional tone of work-related dreams was related to stress and the emotions related to work in waking life. Overall, the findings demonstrate that professional life has a profound effect on dreaming in many individuals—even after years. The next steps would be to study the dream content of work-related dreams and relate these contents to specific characteristics about the jobs, e.g., professional field, hierarchical position and autonomy, etc.

## 1. Introduction

The original formulation of the continuity hypothesis of dreaming [1] simply stated ‘that dreams are continuous with waking life (p. 104).’ The authors added that this continuity may be between dreams and covert behavior, or it may be between dreams and overt behavior, implying that waking-life experience (overt behavior) as well as waking thoughts and fantasies (covert behavior) can be incorporated into dreams. Whereas the theoretical discussion about what aspects of waking-life are more likely to be incorporated into dreams is still ongoing [2,3,4,5,6,7], empirical dream research has focused on studying the factors that might affect the continuity between waking and dreams [8]. 

One of the factors is the time spent with a particular waking-life activity, e.g., sports students dream more often about sports than psychology students [9,10], political science students dream more often about politics than other students [11], and music students dream more often about music than other students [12]. Another example is that students who are driving a lot during the day dream more often about driving [13], and the time spent consuming different types of media is related to the percentage of media-related dreams [14]. A second factor that affects the probability that specific waking-life events are incorporated into subsequent dreams is emotional intensity [15,16], i.e., emotionally intense experiences are more likely to be incorporated. Interestingly, the emotional tone (positive vs. negative) was not associated with the incorporation rate [15]. 

Another factor that affects the continuity between waking and dreaming is the time interval between the waking-life experience and the dream occurrence, e.g., after breaking up with a romantic partner the percentage of dreams including this partner declines [17]. A methodological problem with several studies in this field [18,19,20] is that the temporal references were elicited after the dream was reported, i.e., the participants were asked when the waking-life experience related to the dream occurred. The problem with this retrospective approach is that human memory capacity is limited, i.e., remembering all the experiences and especially thoughts from the previous days is difficult to impossible. On the other hand, carrying out long-term studies to investigate the effect of life events on dreaming after months or years is very time-consuming [21]. That is, empirical research regarding the time factor, especially if longer time intervals are of interest, is not easy. 

Although work is a major part of life for most people in countries all over the world [22], and one would expect—based on the continuity hypothesis of dreaming—that work-related dreams are quite frequent, dream research has rarely studied this topic systematically. The main reason might be that many content analytic studies are carried out in student samples [23,24,25], i.e., individuals that are not yet fully part of the working force. In 442 dreams reported by students, 4.98% were work-related, and the time students spent earning extra money was related to the number of work-related dreams [13], supporting the continuity hypothesis of dreaming. In a sample of 2894 of most recent dreams from adults, 18.0% of the men’s dreams including work topics, compared to 9.3% in women’s dreams—a highly significant gender difference [26]. This study also indicated that work-related dreams are more prominent in non-student samples; this was also found in a sample of adult patients with sleep disorders: 18.39% of 897 dream reports included work-related topics (unpublished data from the author). A gender difference was also found in four independent surveys carried out from 1956 to 2000 [27]. Interestingly, the percentage of persons reporting work-related dreams increased over time, from 18.7% (1956) to 29.9% (2000) in women and from 27.2% (1956) to 37.6% (2000) in men. So far, only two studies [28,29] that focused directly on work-related dreams have been carried out. In the first study, 81 Swiss truck drivers driving on average about 35 h per week completed a dream questionnaire indicating that 16.8% of their dreams were about driving [28]. Work-related stress was associated with more negatively toned driving dreams, whereas long-distance drivers reported higher percentages of driving dreams. In the sample of 87 female hairdressers, work-related dreams were more common in women who were already in the business for a long time [29], but work-related dreams occurred less often if overall life satisfaction was high. Job satisfaction showed a relationship to the emotional tone of work-related dreams [29]. Interestingly, in this sample, the percentage of work-related dreams was quite low (about 4% of all remembered dreams). Out of 80 elderly (75 years old), 22.5% reported work-related dreams, more than 50% with negative emotions, e.g., tools were lost, work could not be completed or was unsuccessful, or feelings of uselessness [30]. The authors interpreted their findings in a way that these dreams reflected the sorrow connected with retirement. One participant, a former shopkeeper, dreamed repeatedly that the goods in his shop are all mixed up and that he is under pressure serving customers, probably reflecting memory problems he had at the end of his professional life in waking [30]. To summarize, the studies on work-related dreams indicate that this type of dream is quite common in the adult population, and that the frequency and emotional tone of these dreams are related to job involvement, job satisfaction and work-related stress levels. 

The aim of the current study was to investigate the frequency and correlates of work-related dreams in a population-based sample. Based on the continuity hypothesis, it was expected that individuals who are currently working dream more often about work than those who are currently not working. Moreover, work-related stress levels should increase the number of work-related dreams, as the waking-life experiences related to work are emotionally more intense. The emotions of the work-related dreams should reflect the waking-life emotions associated with the current or former job.

## 2. Method

### 2.1. Participants

Overall, 1695 persons (960 women, 735 men) completed the online survey entitled ‘Everyday life and dreams’ between 13 April 2020 and 20 April 2020. The mean age of the total sample was 53.84 ± 13.99 years (range: 20 to 96 years). Educational levels were distributed as follows: 0.53% had no degree, 12.80% had 9 years of schooling, 39.62% had O-levels (middle degrees, ‘Realschule’, about 10 years), 23.13% A-levels (‘Abitur’), 30.91% had obtained a university degree, and 3.01% had doctoral degrees. The question about the current work status was answered by 1648 participants: 724 individuals worked full-time (35 h per week or more), 263 individuals worked part-time (15 to 14 h per week), 105 individuals worked less than 15 h per week, and 556 individuals currently did not work. This group was distributed as follows: retired (*N* = 337), long-term leave due to illness and other reasons (*N* = 70), home-maker (*N* = 62), unemployed (*N* = 31), trainee (*N* = 13), student (*N* = 12), voluntary social year (*N* = 1), other reasons for being not employed (*N* = 28), and 2 participants without a current job did not provide details. The time interval since last working was 9.31 ± 7.98 years (*N* = 470, with 2 participants not completing the item and 84 missing values due to technical problems with the filter, i.e., this item was not presented to a subgroup of persons not working).

### 2.2. Research Instrument

A 7-point scale (coded as 0 = never, 1 = less than once a month, 2 = about once a month, 3 = about two to three times a month, 4 = about once a week, 5 = several times a week, 6 = almost every morning) was presented in order to assess overall dream recall frequency. The retest reliability of this scale is high: *r* = 0.85 for an average interval of about 55 days (Schredl, 2004). Five answering options (−2 = very negative, −1 = somewhat negative, 0 = neutral, +1 = somewhat positive, and +2 = very positive) were presented to assess the overall emotional tone of the dreams.

Next, the participants were asked to estimate retrospectively the percentage of work-related dreams they had. The following definition was included: “A work-related dream takes place in your usual work environment and/or includes work-related activities that you deal with in real life. Dreams in which your work colleagues show up but are not related to your work itself should not be included.” The emotional tone of the work-related dreams was—similar to the general emotional tone of dreams—measured via a five-point scale ranging from −2 (very negative) to +2 (very positive).

After eliciting whether the participant is currently employed or not (see participant section), the participants were asked to estimate the emotions about their (former) employment: “How would you describe your emotions about your (former) work in general? −2 = ‘I don’t/didn’t like my work at all’, −1 = ‘I don’t/didn’t like my work’, 0 = ‘Neutral’, +1 = I like/liked my work, and +2 = ‘I like/liked my work very much’.” Lastly, work-related stress was elicited: “How stressful do/did you experience your (former) work in general?’ 0 = not stressful at all, 1 = not very stressful, 2 = neutral, 3 = rather stressful, and 4 = very stressful”.

### 2.3. Procedure

Within the online panel www.wisopanel.net, persons with an interest in online studies and with heterogenic demographic backgrounds are registered. At the time of the study, 14,277 individuals were in the database. Almost all participants were German citizens, with a few exceptions of Austrian and Swiss citizens. All registered persons received an email with the link to the study entitled ‘Everyday life and dreams’ (The questionnaire was in the German language). The participation was voluntary and unpaid. The study was carried out following the rules of the Declaration of Helsinki of 1975 (https://www.wma.net/what-we-do/medical-ethics/declaration-of-helsinki/), as revised in 2013. In Germany, this type of study—asking healthy individuals to participate in an online survey on a completely voluntary basis—does not require ethical approval. This was confirmed by the ethics committee of the University of Mannheim.

Statistical procedures were carried out with the SAS 9.4 software package for Windows. As the percentage variables (the percentage of work-related dreams) were not normally distributed, they were categorized (see the results section). The categorized variables were treated as ordinal variables. Ordinal regressions were used to analyze the effect of waking-life variables on dream variables controlled for age, sex, education and dream recall frequency. All variables were entered simultaneously. Effect sizes were computed based on Chi-Square values according the formula given by Cohen [31]. Throughout the manuscript, means ± standard deviations are presented.

## 3. Results

The dream recall frequency distribution was as follows: never (8.69%), less than once a month (18.79%), about once a month (8.87%), about two to three times a month (13.36%), about once a week (18.62%), several times a week (22.99%), almost every morning (8.69%), and three missing values. The mean of the percentage of work-related dreams was 17.56 ± 22.86% (*N* = 1691). The distribution of the categorized variable is depicted in Table 1. Overall, more than 70% of the participants reported that they had had work-related dreams. The emotional tones of work-related dreams are shown in Table 2; there is a slight shift towards more negative emotions (mean: −0.09 ± 0.87). For the subgroup of participants completing both scales on the general emotional tone of dreams (mean: 0.03 ± 0.81, *N* = 1175) and the emotional tone of work-related dreams, the emotional tone of work-related dreams was significantly more negative (mean: −0.10 ± 0.87, *N* = 1145; Wilcoxon Signed Ranks test: *z* = −5.0, *p* < 0.0001, effect size = −0.298).

The percentages of work-related dreams and the averaged emotional tone of work-related dreams are presented in Table 3 for the four groups (currently working and currently not working). Interestingly, 23.20% of the participants with full-time jobs did not report any work-related dreams. The emotional tone regarding current or former work was on average positive: 0.95 ± 1.05. The mean stress level amounted to 2.31 ± 1.06, indicating moderate stress levels. Interestingly, the correlation between these two scales was relatively small: *r* = −0.154, *p* < 0.0001, *N* = 1675. The ordinal regression for the total group indicated that working vs. not working had a significant effect on the percentage of work-related dreams, with an effect size of *d* = 0.240 (see Table 4). In addition, higher work-related stress levels were also related to more work-related dreams (effect size: *d* = 0.329), whereas the emotional attitude toward the (former) job had no effect. Moreover, men dreamed more often about work (*d* = 0.166), whereas age and educational level was not associated with the percentage of work-related dreams. The possible confounder of dream recall frequency was strongly associated with the percentage of work-related dreams. Whereas working vs. not working did not affect the emotional tone of work-related dreams, the emotional attitude towards work (*d* = 0.500) and the work-related stress levels (*d* = 0.554) were associated with the emotional tone of work-related dreams (see Table 4). Moreover, men reported more negative work-related dreams than women (*d* = 0.160) and individuals with lower educational levels reported more negatively toned work-related dreams (*d* = 0.163).

For the subgroup of participants currently working, the amount of work was related to the percentage of work-related dreams (with a very small effect size of *d* = 0.120), the effect of work-related stress levels was much stronger (*d* = 0.353) (see Table 5). Regarding the emotional tone of work-related dreams, the overall pattern is similar to the pattern found in the total sample; the effect of lower educational levels was even stronger (*d* = 0.259). As expected, the length of the time interval between completing the questionnaire and the last employment was related to the percentage of work-related dreams. Stress levels experienced in the former job were also correlated with the percentage of work-related dreams (see Table 6). Whereas there was no gender difference regarding the percentage of work-related dreams in the group currently working, men reported more dreams about former work than women (*d* = 0.356) (see Table 6). The emotional tone of the dreams related to the former job was again associated with the emotions (effect size: *d* = 0.401) and stress levels (*d* = 0.481) associated with the job. Interestingly, there was an age effect, i.e., older people reported more positively toned work-related dreams (*d* = 0.325). 

## 4. Discussion

The findings indicate that every fifth dream is related to current or previous work. Individuals who currently work dream more often about work; jobs experienced as more stressful are more likely to affect dream content. The emotional tone of work-related dreams was related to stress and the emotions related to work in waking life. Thus, the current findings support the continuity hypothesis of dreaming.

Several methodological issues have to be considered. First, even though the sample showed a large age range and different educational backgrounds, it was self-selected: these are (A) individuals interested in online surveys and, therefore, registered in the panel; and (B) these individuals are ones who are interested in dreams, since they chose to participate in this particular dream-related online survey. Comparing the dream recall of the present study to representative samples [32], the results indicate that high recallers are overrepresented and, therefore, it was important to include dream recall frequency as a covariate in the analysis. Similarly, higher educational levels are overrepresented [33], so education was also introduced as a possible confounder into the regression analyses. 

The data showed a strong relationship between the retrospectively measured percentage of work-related dreams and dream recall frequency. One explanation might be that persons with very low dream recall might have difficulties remembering what they dreamed about (because their dreams are so rare); some evidence supporting this idea was presented by Schredl [34]. On the other hand, it might be possible that a third variable, for example openness to experience, explains this relationship. Openness to experience is related to dream recall frequency [35], and possibly to the closeness between waking life and dreaming [36], i.e., the likelihood of waking-life events showing up in dreams. In any case, it was important to include dream recall frequency into the analyses as a possible confounder to control for such effects. The validity of retrospectively eliciting the occurrence of specific dream themes was demonstrated by Erlacher and Schredl [9] and Schredl and Erlacher [10], who compared sport dreams coded in diary dreams with the retrospective format, and found similar percentages of sports dreams. Moreover, the mean percentage of work-related dreams of about 18% fits in nicely with the percentage of work-related dreams (also about 18%) found in 897 dreams of patients with sleep disorders (from the unpublished data of the author).

The retrospectively measured percentage of work-related dreams of about 18% indicates that this dream topic is more prominent that topics like sports [37], politics [37], music [38] or media [14], with mean percentages ranging from 4% to 6.5%. Interestingly, the frequency of erotic dreams, which was also about 18% [39], is comparable, highlighting the notion that work is a major part of adult human life. Interestingly, the emotional tone of work-related dreams was slightly but significantly more negative compared to the participants’ general emotional dream tone. In dreams including leisure time activities like sports, music and erotic activities, the emotional tone was more positive than the general dream tone [37,39,40], indicating that work-related dreams might reflect the stressful aspects of work (see below).

As expected, individuals who are currently working dreamed more often about work than non-working individuals; a finding that is in line with the continuity hypothesis [8]. Moreover, working more hours per week was also related to more work-related dreams, as were the shorter time intervals between completing the questionnaire and having had a job in individuals who are currently not working. This replicates previous findings [13,20] and supports the hypothesis that time spent with an activity in waking life is related to dream incorporation, and the time interval between waking-life experience and dream occurrence are factors modulating continuity [8]. On the other hand, the effect of working vs. not working was not very large, mainly because non-working individuals also report a high percentage of work-related dreams (about 15%). This indicates that work is a topic that has long-lasting effects on dreams, especially in men. Gender differences regarding the occurrence of work-related dreams were reported previously [26,27], and are especially pronounced in older individuals (60 years and older), with far more men reporting work-related dreams [27], supporting the idea that professional life is very important for men, possibly more important than for women [41]. The findings also indicated that even individuals working full-time did not dream about their job. One explanation might be low dream recall frequency (see the effect of dream recall frequency in the regression analysis); that is, the rare dreams might deal with personal issues and not with work-related topics. In addition, it would be interesting whether this finding might also be explained by the professional field of the dreamer, as previous research has shown that ‘focused cognitive activities’ like reading, writing and arithmetic occur very rarely in dreams [13,42]. The Social Simulation Theory of dreaming postulates that dreams select for social topics [43] and, thus, some aspects of professional life might be under-represented in dreams.

Whereas the emotional quality related to work in waking was not associated with the frequency of work-related dreams, the stress levels of current work and of former work were related, indicating that intensity and not emotional tone (e.g., a preference of negative experiences to be incorporated into dreams) is of importance as a moderating effect regarding the continuity between waking and dreaming [15].

The emotional tone of work dreams was—as expected—related to stress levels and the emotional quality associated with work in waking, supporting the notion that dreams are not only continuous to waking life on a thematic level but also on an emotional level [44]. This was also found for work-related dreams in truck drivers [28] and hair dressers [29]. It was also interesting to see that this emotional continuity was also found in persons who no longer work (an average of about 9 years since the last job), i.e., stressful and negatively evaluated work has a long-lasting effect on dreaming. This is in line with the findings of negative work-related dreams which were reported by Achte and Malassu [30], and also possibly reflecting stress with the former job or issues related to retirement. Another interesting finding was that low education was related to more negatively-toned work-related dreams in persons who are currently working. It would be interesting to study whether this is related to professional hierarchy (more or less autonomy within the workplace). Lastly, age was related to more positive work-related dreams, i.e., retired persons who might look back on their long professional lives might be more relaxed in their dreams if work-related topics come up.

## 5. Conclusions

To summarize, the findings of the present study clearly indicate that work-related dreams reflect waking-life experiences regarding work, including the stressfulness and emotional aspects. The next steps would be to study the dream content of work-related dreams, what kind of work-related dream activities are positive, e.g., completing a difficult task within the dream or pleasant social encounters, or negative, e.g., failing to do something or being bullied, etc. Another interesting aspect would be to elicit the profession of the dreamers, including specific characteristics about the job, e.g., hierarchical position and autonomy, etc. It would also be very interesting to study the dreams of shift workers, as shiftwork has a very strong effect on sleep [45]. As professional life is a major part of modern human life, studying work-related dreams can help us to understand its effect on the inner world of the individual, e.g. burn-out syndromes, and also—on a theoretical level—help to clarify the relationship between waking and dreaming, i.e., the factors that affect this continuity.

## Figures and Tables

**Table 1:** Percentage of work-related dreams (retrospective estimates; *N* = 1691).

Category	Frequency	Percentage
more than 60%	121	7.16%
40.01% to 60.00%	126	7.45%
20.01% to 40%	211	12.48%
10.01% to 20%	197	11.65%
5.01% to 10%	217	12.85%
0.01% to 5%	327	19.34%
0%	492	29.10%

**Table 2:** Overall emotional tone of work-related dreams and the general emotional tone of dreams.

	Emotional tone of work-related dreams (*N* = 1197) ^1^		General emotional tone of dreams (*N* = 1531)	
Category	N	Percent	N	Percent
Very positive (+2)	28	2.34%	36	2.35%
Somewhat positive (+1)	276	23.05%	431	28.15%
Neutral (0)	492	41.10%	689	45.00%
Somewhat negative (−1)	358	29.91%	351	22.93%
Very negative (−2)	43	3.59%	24	1.57%

Note: ^1^ two values were missing.

**Table 3:** Means and standard deviations for the retrospectively estimated percentages of work-related dreams and the overall emotional tone of work-related dreams.

Group	Percentage of work-related dreams	Emotional tone of work-related dreams
Working full-time (35 h per week or more)	19.74 ± 22.72% (*N* = 724)	−0.15 ± 0.85 (*N* = 556)
Working part-time (15 to 34 h per week)	15.99 ± 20.62% (*N* = 263)	−0.06 ± 0.86 (*N* = 199)
Working part-time (less than 15 h per week)	16.09 ± 21.99% (*N* = 104)	0.05 ± 0.86 (*N* = 77)
Currently not working	15.26 ± 23.19% (*N* = 553)	−0.07 ± 0.91 (*N* = 335)

**Table 4:** Ordinal regression analysis for the categorized (ordinal) percentages of work-related dreams (retrospective estimates) and the overall emotional tone of work-related dreams (total sample).

	Percentage of work-related dreams (*N* = 1624)			Emotional tone of work-related dreams (*N* = 1162)		
Variable	SE	χ^2^	*p*	SE	χ^2^	*p*
Age	0.0253	0.8	0.3703	0.0236	0.5	0.4962
Gender	−0.0841	11.1	0.0009	−0.0855	7.4	0.0064
Education	0.0473	3.6	0.0590	−0.0853	7.7	0.0057
Dream recall frequency	0.3658	190.8	<0.0001	0.0155	0.2	0.6171
Currently working vs. not currently working	0.1323	23.1	<0.0001	−0.0017	0.0	0.9593
Emotions related to work	−0.0020	0.0	0.9384	0.2707	68.3	<0.0001
Work-related stress levels	0.1666	42.7	<0.0001	−0.2917	82.8	<0.0001

SE = standardized estimates.

**Table 5:** Ordinal regression analysis for the categorized (ordinal) percentage of work-related dreams (retrospective estimates) and the overall emotional tone of work-related dreams (only for individuals currently working).

	Percentage of work-related dreams (*N* = 1087)			Emotional tone of work-related dreams (*N* = 830)		
Variable	SE	χ^2^	*p*	SE	χ^2^	*p*
Age	0.0301	0.9	0.3420	−0.0346	0.8	0.3716
Gender	−0.0115	0.1	0.7148	−0.0753	3.9	0.0483
Education	0.0553	3.8	0.0661	−0.1356	13.7	0.0002
Dream recall frequency	0.3441	115.6	<0.0001	0.0069	0.0	0.8533
Working hours per week (three groups)	0.0627	3.9	0.0497	−0.0468	1.5	0.2260
Emotions related to work	−0.0136	0.2	0.6578	0.2960	56.8	<0.0001
Work-related stress levels	0.1792	32.8	<0.0001	−0.3168	65.9	<0.0001

SE = Standardized estimates.

**Table 6:** Ordinal regression analysis for the categorized (ordinal) percentage of work-related dreams and the emotional tone of work-related dreams (only for individuals currently not working).

	Percentage of work-related dreams (*N* = 449)			Emotional tone of work-related dreams (*N* = 269)		
Variable	SE	χ^2^	*p*	SE	χ^2^	*p*
Age	0.0377	0.5	0.5008	0.1962	6.9	0.0085
Gender	−0.1974	13.8	0.0002	−0.0427	0.4	0.5376
Education	0.0101	0.0	0.8360	0.0179	0.1	0.7795
Dream recall frequency	0.4330	68.3	<0.0001	0.0033	0.0	0.9588
Time interval to last employment	−0.0950	3.2	0.0373 ^1^	−0.0444	0.4	0.5082
Emotions related to work	0.0251	0.2	0.6178	0.2158	10.4	0.0013
Work-related stress levels	0.1032	4.3	0.0375	−0.2481	14.7	0.0001

SE = standardized estimates, ^1^ one-tailed.
